# Optimism and Health: Resource or Delusion

**DOI:** 10.1093/geroni/igab046.1568

**Published:** 2021-12-17

**Authors:** William Chopik

**Affiliations:** Michigan State University, East Lansing, Michigan, United States

## Abstract

There is a general, widely-held belief that optimism is always a good thing. While there is much previous research suggesting that optimists enjoy several health and wellness benefits, there is also a large body of research suggesting that optimism is not always advantageous. Examining how optimism develops and changes across the lifespan may give us insight into how we use optimism and allow us to determine if and when optimism is helpful or maladaptive. In this talk, I will review evidence debating the efficacy of optimism, as well as examine how optimism develops across the lifespan. I also discuss how life events may or may not impact the developmental trajectory of optimism. Lastly, I address currently unanswered questions and emphasize the contextual nature of optimism’s advantages. Ultimately, being persistently optimistic about the future is a nearly universal human trait. But the exact contexts in which this positive thinking is helpful--if ever--is an intriguing question that speaks to how we think about ourselves, how we think about others, and how we think about our many possible futures.

